# Use of the Letheon

**Published:** 1847-10

**Authors:** Joseph Taylor


### *On the Use of Letheon, by* JOSEPH TAYLOR, D. D. S.

MAYSAILLE, Oct. 10th, 1847.

TO THE EDITORS OF THE DENTAL REGISTER:

*Gentlemen*:—Since the propriety of using Dr. Morton's Letheon for alleviating pain during Surgical operations has excited so much interest in the public mind—and as the propriety of administering it indiscriminately is as yet questionable, it would seem highly necessary that we should have some way of discriminating how and to whom it should be administered. Although men of eminence in the medical profession have thrown much light upon the subject, and have given some excellent rules by which those who administer it should be governed—yet there being such diversity of effects produced by the same causes, and the symptoms and considerations laid down as our guide so often failing—I have thought it would be of interest to some to give publicity to a few cases which have come within my observation. Notwithstanding upon a very large majority of persons the same symptoms and effects are produced, yet the following will show how difficult it is to discriminate upon cases which are more diversified.

A lady of sanguine temperament called on me a few months since, for the purpose of having a tooth extracted under the influence of the vapor, requesting me to give her as little as possible to produce the desired effect. Accordingly, after inhaling a very small portion, she observed that she felt its influence. I then separated the gum from one side of the tooth, and inquired if she felt any pain. She answered in the negative; and after the gum was entirely separated, I interrogated her again; her answer was still the same, observing that I must be in haste, that she felt it passing off. I immediately removed the tooth, and, to her great delight, without any suffering whatever. This is the most favorable case out of about three hundred and fifty which have occurred in my practice; it required less of the vapor, while its duration gave me sufficient time to extract two teeth—at the same time, leaving her perfectly conscious, conversed as usual without exhibiting any of the syptoms which occur generally. In contrast with the above, I would relate the case of Dr. G., a gentleman of a high nervous and bilious temperament, who prevailed upon me (much against my will) to administer it to him. After inhaling it for about forty seconds, his breathing became long, full and easy, and in a few moments his face became somewhat florid, the pupil of his eye dilated, and exhibited some agitation, when with one stretch of his arm he sent me, Letheon and all some distance across the room. I immediately presented myself to him again and with some difficulty succeeded in removing the tooth, causing much suffering, which continued some time after the effects of the Leatheon passed off; he was unconscious of every thing except the pain consequent upon the operation, which he described as being excruciating. In this instance it would seem that the nerves of the brain being very susceptible, became over excited; consequently the blood terminated to the head before it took effect upon the nerves in general. These two cases present the extreme, both for and against its use; however, neither of the cases would apply in general, the most usual result being a relaxation of the whole muscular system and complete insensibility to every thing, which continues about thirty seconds.

I have thought it requisite to introduce a case to show that the Letheon can be administered to the aged as well as any.

About five months ago I called on a lady about sixty-eight years of age, who had been laboring under pulmonary disease for several years, for the purpose of extracting a tooth. She requested me to put her under the influence of the vapor; I hesitated, but as it was the direction of her physician, I immediately proceeded to perform the operation, which was done without giving her any pain whatever. The vapor appeared to have salutary effects. I had forgotten to name that she had been confined to her bed for several months. She rested the following night better than usual. The next morning she requested me to give it to her again, as its influence was so very pleasant, which I did with the same success, and all passed off without any bad effects upon her system.

And another case occurred, dissimilar to either. A gentleman who inhaled it, and upon whom it had the most happy effect, causing the most thrilling sensation of pleasure, submitted cheerfully to an operation which was entirely unknown to him until he awoke from his reverie, when he assured me that he did not know that I was present. A day or two afterwards I administered it to him again, producing the same pleasant sensation, but an unwillingness to have me operate ; since when he has inhaled it two or three times, always producing the same symptoms, yet evading me when I attempted to approach. Each time it had less effect upon him ; the last time it passed off almost as soon as he ceased inhaling it. Others again have taken it seven or eight times, always with success ; yet I could perceive the more frequently it was inhaled, the more it took to produce the desired effect, and would pass off more rapidly. It is but seldom that a person of a very excitable nervous temperament can be brought sufficiently under its influence so as to prevent all pain consequent upon a severe operation, though it may mitigate it, and in some instances it would be admissible to give it to such. Yet if we do not succeed by the first effort, it is but seldom that a repitition will produce the desired result. There are many other cases which I could enumerate, but as I have already trespassed upon your columns, I would close by referring to one of interest, the import of which is set forth in a note from Dr. McGarrough, the Surgeon who performed the operation, which I enclose to you.

Yours, &c.,

JOS. TAYLOR.

Greenfield, Oct. 11th 1847.

Dr. Joseph Taylor :

*Dear Sir :*—I received yours of the 4th inst., in which you expressed a wish to know how Mrs. Gulliford got along, to whom you administered the Letheon when I amputated her breast.

I am happy to have it in my power to state that I have never seen a wound of the same magnitude heal so completely by the first intention. I enquired of her whether she felt any pain during the operation. She said she felt none, but thought she was contending in argument with some person. She had none of that prostration and nervous debility which often follow operations of magnitude. She told me that she supposed that there never was a woman who had lost a breast with as little suffering. This is a century of invention. Thoughts are carried along cold wires with the velocity of lightning. Breasts and limbs are amputated without pain.

Yours respectfully,

T. McGARROUGH.

